# 

**Published:** 1888-01-07

**Authors:** 


**NOTICES.**

THE scale of charges for small prepaid Advertisements—Situations Wanted, Situations Vacant, &c., &c., is as follows:—

| | s. | d. |
|---|---|---|
| One insertion of three lines | 1 | 0 |
| Three „ „ „ | 2 | 6 |
| Per line afterwards | 0 | 6 |

*A line averages ten words.*

All copy for Advertisements must be sent to A. P. WATT, 2, PATERNOSTER SQUARE, E.C., by first post on the Tuesday *preceding* publication.

**All communications respecting editorial matters** should be addressed to the Editor, 38, Old Jewry, Cheapside, E.C.

### "THE HOSPITAL" ALMANAC FOR 1888.

WE this week publish, for the first time, an Almanac, which will be presented gratis with every copy of this week's HOSPITAL The Almanac was compiled by F. C. Carr-Gomm, Esq., chairman of the London Hospital, and contains full particulars of the metropolitan hospitals in the year of Jubilee, classified as "Endowed," "Chest," "Children's," "Lying-in," "Women's," and "Other Special Hospitals." The results of the collections on Hospital Sunday and Saturday last year, with the cost of raising the former fund; the number of institutions benefited, and the dates on which these collections will be held in 1888 are first given. Then the address, date of foundation, resident management, committees with days and hours of meetings, matrons and nursing staff, visiting and medical staff with days and hours of attendance, the resident medical staff, the total and average number of beds occupied in 1886, the cost of each in-patient per week, the sources of income, qualification of governors, cost of endowing a bed, and particulars of the medical schools Samaritan funds, convalescent homes, and other special features of every hospital, all arranged in such a form as to make them easily available for reference and comparison. This Almanac would no doubt be found of great value if suspended in workshops, police stations, and Board schools throughout the metropolis, as well as at the hospitals, where it is likely to find a place, not only in the wards, but also in the out-patient departments. The Almanac states that the metropolitan hospitals require an additional income of £100,000 to enable the managers to maintain all the beds constantly occupied. As Portia said to Shylock, so we may say to Londoners, " 'T'were well you do so much for charity."

Copies of the Almanac (which is copyright and has been entered at Stationers' Hall) may be had separately on application to the publisher, at the following rates: If printed on stiff paper, 6*d*. per copy, or 5*s*. per dozen; if mounted on linen, with rollers and varnished, at 1*s*. 6*d*. each copy, or 1*s*. 10*d*. post free; or 15*s*. per dozen. The Almanac can be ordered through any bookseller or newsagent.

### AMUSEMENTS WITH PROFIT FOR ALL AGES.

**Rules.**—All answers must be addressed to the PRIZE EDITOR, THE HOSPITAL, 38, Old Jewry, Cheapside, London, E.C., not later than the *first* post on Thursday next. The full name and address must in all cases be given, but a *nom de plume* may be added for publication. Only one side of the paper must be written on.

### FOR MEN AND WOMEN.

### Work Competition.

I. A PRIZE OF THREE GUINEAS will be given to the competitor who sends in the best specimen of painting, which may take the form of a picture, panel, screen, flower-pots, etc., or illuminations.

II. TWO GUINEAS will be given for the best dressed doll or other piece of work suitable for presentation to the inmates of the hospitals.

III. ONE GUINEA will be given for the best scrap-book.

All Contributions for this competition must be sent in by January 7th, 1888, addressed "Work Competition," care of the Editor, The Lodge, Porchester Square, London, W.

Results of above Competition will appear on January 14.

### Acrostic Competition.

A PRIZE OF ONE GUINEA will be given to the competitor who gains the highest number of marks for best original acrostics during the ensuing quarter. All acrostics sent in for this competition will be credited according to merit, twelve marks being the highest score given for one acrostic.

A PRIZE of HALF-A-GUINEA to be given to the competitor who gains the highest score for solution of acrostics set, including the one inserted on November 26th. One mark only will be given to the competitors who solve all the lights of each acrostic.

These competitions to alternate weekly.

This week credit will be given for solution of the following :—

No. 4. DOUBLE ACROSTIC.

"Alas, forgotten or remembered still,
'Mid joy or sorrow, fate will work its will."

1. "He shifted his trumpet, and only took snuff."
2. "And here a chancellor in ——."
3. "Many a hand's on a richer hilt,
But none on a steel more ruddily gilt."
4. "And many a banner shall be torn,
And many a knight to earth be borne;
And many a sheaf of arrows spent,
'Ere Scotland's King shall cross the ——."
5. "Give a direction for this merry bond"
6. "Mighty sailor, this is he,
Was great by land as thou by sea."
7. "Heap on more wood, the wind is chill,
But let it whistle as it will,
We'll keep our merry Christmas still."

The author of the quotations must be given.

No. 3. DOUBLE ACROSTIC.

**Answer.**

| | | |
|---|---|---|
| R | om | P |
| O | rdea | L |
| A | ngerea | U |
| S | unbea | M |
| T | i | P |
| T | | U |
| U | naske | D |
| R | ochefoucaul | D |
| K | ep | I |
| E | de | N |
| Y | oun | G |

Correct answers have been received from Pasteur, Gretta, Brownie, Shark, Dunce, Ellice, Aralc, Mater, Lady Clara, Canice, Gretta, Reldas, Condorcet.

### II. Word and Literary Competition.

Commenced November 5th, 1887; ends January 28th, 1888.

Three prizes of one guinea, 15*s.* and 10*s.* 6*d.* will be given each quarter to the three competitors who obtain the highest number of marks: (1) by making the largest number of words derived from the word or phrase set for anatomical dissection; or (2) for the best answers to (6); or (3) by (1) and (2) combined.

(A) THE WORD COMPETITION.

We shall give the newspapers for dissection during this quarter, that for this week being

"DAILY NEWS."

OBSERVE.—Proper names, abbreviations, foreign words, words of less than four letters and repetitions are barred. Nuttall's *Standard* dictionary only to be used.

### Results of Eighth Week.

THE LANCET.—E. E. (98), Reldas (96), Tabs (94), Brisbane (92), Gretta (89), Ellice (87), Skye (87), K. M. (46), Brownie (33).

### Letters from Across the Seas.

*Gretta* sends the following from Kimberly, S. Africa:—I wrote to you on my arriving at Graham's Town after my trip to Port Elizabeth, which ended so unfortunately as regards any chance of my coming home. On reaching Graham's Town I immediately waited on Navvy C——, and it ultimately resulted in his son obtaining 20*l.* for our joint benefit to proceed to Orange River to start a hop and ginger beer manufactory half-way between there and here. We left Graham's Town on Wednesday night and arrived at Orange River Station on Friday morning, thoroughly tired out with the travelling, and after dinner I retired to a neighbouring tent to have a nap. I slept until six o'clock, and on my awaking failed to discover C ——, jun., anywhere. It seems he had cleared off during the time and taken every shilling with him. I did not find this out until about 9 p.m., and you can imagine my consternation especially as I had not a penny, and knew no one in the place, and over four hundred miles from the bay. However, I went back to the tent and managed to get a resting-place for the night, and in the morning went and saw the engineer, who said that contracts for the line were not ready. In the evening of the same day I determined to make for the fields, and joined company with another man who was going there penniless but with good testimonials, the only funds in hand being ten shillings I had raised on a pair of boots. We arrived late that evening at a farmhouse, and seeing a light, knocked and applied for accommodation (a connection of a missionary); the gentleman made us up a bed on the floor of his parlour, and gave us some bread and some milk (brod and dick merelk the Dutchman says) for an early breakfast. After thanking him, not daring to offend by offering payment, we started and overtook some waggons on the same road, and leaving our kits with them, promised to await them at the next out-span about fourteen miles further on, where we arrived about 6 p.m. There were any amount of Dutchmen only too delighted to see Englishmen on foot. How we passed that night I cannot describe fully enough for you to understand. We had left our waistcoats with our things on the waggons, and we neither of us wore any underclothing. The waggons did not arrive till next morning, and if we had not kept moving about and smoked the whole night we should have died from the cold. The Dutchmen sat round their fires drinking coffee and laughing at us. The morning brought the waggons, and we took our things and started. About midday we passed the lot of Dutchmen who sneered at us, and I, wild with their conduct of the previous night, could not pass them without saying something. We had a few words, and I wanted to fight them one at a time, but, on their declining, told them "they had better not laugh at the English yet awhile." We arrived here Friday, and I began work for a Mr. D—— on the following Monday, at £3 a-week, but the living is very dear. Do not think I am any the worse for my trip; I never felt better in my life.

I am pleased to tell you I am again, after waiting five weeks for the appointment, time-keeper on "the fifteen-mile length." I should have written last week, but there was not a piece of writing paper or envelope in the whole camp. I have seven camps in my length, which is two miles 68 chains. The entire way three times a day it would be seventeen miles daily, but the last two gangs I only visit once a-day. Wages are £12 10*s.* a-month, and I have one nigger for my servant. The first night I was here I had a leg of mutton stolen from the tent and some bread while I was away with the ration waggon, which brings food two or three times a-week. If any of the men want anything they have to come to me for a ticket, which is in the same form as a cheque, so I have plenty to do, as one gang alone has fifty men in it. I think the boss is rather taken with me because I take an interest in the work. I have power to discharge any ganger or labourer (black or white) on my length; in fact, I am the inspector, pro tem., in addition to my other duties.

When this contract is finished I shall shift to Natal or Worcester. I am now getting £12 10*s.* per month, which would be very good were it not for the "Tommy shop system." We have to purchase all rations from the ration waggons, who pay 20 per cent. to the contractors on all goods sold, so the consequence is they charge awfully high. I will give you an idea: Mutton, 7*d.* per lb.; corned beef, 3*s.*; potatos, 6*d.*; flour, 6*d.*; coffee, 2*s.*; tea, 3*s.*; white bread, 1*s.* per loaf; sugar, 6*d.*; matches, 1*s.* per dozen; three very small sticks of cavendish, 1*s.*; fruit salt, 4*s.*, a very small bottle onions, 6*d.*; eggs, 3*d.* each; bacon, 1*s.* 6*d.*; cheese, 2*s.*

### THE CHILDREN'S CORNER.

PRIZES.
FOR MOST CORRECT ANSWERS TO PUZZLES.

This Commenced October 1st, 1887.
One Pound a Month.
FOR BEST LITTLE LETTERS.
Ten Shillings per Quarter.
A PRIZE OF THREE GUINEAS
to the Competitor who shall have sent in the largest number of correct or best answers during the twelve months.

### Puzzles—First Week.

MONTHLY COMPETITION.

No. 1. TRANSPOSITION.

Mighty as the deep we are,
Lighter than its summer spray.
Yet peace and war are in our power,
As forth we fly upon our way;
And now transpose, a weapon, see
No use for it in times of peace.
For action made, it rusts in ease,
And shines in combat gallantly.

No. 2. BIOGRAPHICAL ACROSTIC.—1. A town in Gloucestershire. 2. A battle won by Alphonso over five Moorish kings. 3. The Latin for king. 4. A well known opera of Rossini's. 5. A King of Norway, Sweden, and Denmark who was deposed and settled in Pomerania. 6. The founder of the Stoic sect. The initials give the name of a celebrated general, and the finals one of his conquests.

No. 3. ENIGMA.

Soon as I'm made I'm sought with care,
For one whole year consulted;
That time elapsed, I'm thrown aside,
Neglected and insulted.

No. 4. ENGLISH TOWNS ENIGMATICALLY EXPRESSED. 1. Fresh and to inter. 2. Air and three-fifths of to be vexed. 3. A celebrated painter. 4. A flower and a harbour.

### Results of Fourth Week Puzzles.

No. 13. HISTORICAL CHARADE. War-wick.
No. 14. DECAPITATIONS. Neva, eva, shovel, hovel.
No. 15. ENIGMA. Mince-pie.
No. 16. DOUBLE ACROSTIC.

| | | |
|---|---|---|
| H | er | B |
| O | liv | E |
| L | arkspu | R |
| L | obste | R |
| Y | esterda | Y |

*All right*—A. C. K., Tim, Scrooge, Gem, Excelsior Jumps, A. G. S, McCulloch. *Three right*—Fern, Skye, Ossian, Pepita, Happy Eliza, Plummy, Fluff, Judy. *Two marks each*—M. S. W., Military Cadet, Betty Burke, Poodle.

### Letters.

*Three marks each*—Gem, Skye. *Two marks each*—Fern, Ossian.

### Results of Quarterly Letter Competition.

This week we award the letter prize of 10*s.* to *Gem* (Master G. E. Maddock, Porlock, Taunton), who has gained the highest score (35 marks) for letters during the past quarter. For the future the letter competition will be discontinued, and "Letters from across the Seas" will be published instead. We shall be glad to receive contributions to this column from readers who may have letters of general interest to forward, *vide* literary competition.

**Notice to all Correspondents.**—N.B.—All letters referring to this page, which do not arrive at 38, Old Jewry, Cheapside, London, E.C., by the *first post on Thursdays*, and are not addressed PRIZE EDITOR, will in future be disqualified and disregarded.

No one will be permitted to win the Monthly or Quarterly Prizes twice in any one year, the annual prizes being offered especially to prevent this.

